# Monitoring of *Candida* biofilm inhibition by *Galenia africana* using real-time impedance-based technology

**DOI:** 10.22034/cmm.2024.345240.1541

**Published:** 2024-11-16

**Authors:** Pedro Miguel dos Santos Abrantes, Keith Chesnay Stuurman, Jeremy Arthur Klaasen, Charlene Wilma Joyce Africa

**Affiliations:** 1 Maternal Endogenous Infections Studies (MEnIS) Research Laboratories, Department of Medical Biosciences, University of the Western Cape, Bellville, South Africa; 2 Plant Extract Laboratory, Department of Medical Biosciences, University of the Western Cape, Bellville, South Africa

**Keywords:** *Candida*, Fluconazole resistance, *Galenia Africana*, Medicinal plants, xCELLigence system

## Abstract

**Background and Purpose:**

Yeasts of the *Candida* genus are responsible for localized and disseminated infections, especially in immunocompromised populations. These infections are exacerbated by the rapid increase in drug-resistant strains, which limits treatment options and increases patient morbidity and mortality. Therefore, the utilization of easily accessible natural products as alternatives to conventional medicines has gained interest. South Africa is home to a rich biodiverse natural flora of which many are known for their antimicrobial activity, including the antifungal effects of their plant extracts. *Galenia africana* (kraalbos) is a local indigenous plant found to have various traditional uses, including the treatment and prevention of various human infections.

**Materials and Methods:**

In this study, the activity of *G. africana* against *Candida albicans* and *Candida glabrata* preformed biofilm formation and its antibiofilm activity were tested using the xCELLigence system, which monitors biofilm formation in real time using impedance.

**Results:**

Presence of *G. africana* resulted in a dose-dependent decrease in *Candida* biofilms and was found to be effective in the prevention of *Candida* biofilm formation and disruption of the existing *Candida* biofilms.

**Conclusion:**

The xCELLigence impedance-based system proved to be an effective tool for medication screening. To the best of our knowledge, this is the first reported study to use real-time monitoring of a medicinal plant on microbial biofilm formation.

## Introduction

*Candida* species are among the most prevalent aetiologic agents in human invasive infections, with a reported mortality rate of 40-51% [ 1 , 2 ]. Immunocompromised patients and those hospitalized in intensive care units with horizontal transmission are at a higher risk of invasive candidiasis (IC) [ 3 - 5 ].

Although *Candida albicans* has been implicated in the majority of IC cases, the incidence rate of non-albicans *Candida* (NAC) species is rapidly rising, with *Candida glabrata* reported as the second most common cause of candidemia after *C. albicans* [ 6 - 8 ], with morbidity and mortality rates of 40-60%. [ 9 ].

Currently, only three classes of medications are approved for the treatment of systemic fungal infections [ 10 - 12 ]. Azoles have been widely used as the first-line treatment of IC [ 13 - 15 ], but several reports of azole-resistant strains of *C. albicans* and NAC species have emerged, and another major antifungal class, echinocandins, is now used as first-line therapy for the treatment of IC in many countries [ 15 , 16 ]. Most *Candida* species are susceptible to echinocandins and amphotericin B, although a high incidence rate of *C. glabrata* resistance against echinocandins has been reported [ 16 , 17 ], usually accompanied by cross-resistance to azoles [ 18 , 19 ] and reduced sensitivity to amphotericin B [ 20 ]. Medication resistance has been attributed to long-term exposure to antifungal medications [ 3 , 5 , 21 , 22 ] and biofilm formation [ 23 ].

Sessile cells (within the biofilm) differ from planktonic (free-living) populations [ 24 ] in showing higher antifungal resistance levels [ 25 ]. *Candida* biofilms are enclosed in an extracellular matrix (ECM) of carbohydrates (e.g., polysaccharides), proteins, and DNA [ 26 , 27 ]. The ECM of the *Candida* biofilm contributes to pathogenicity by increasing medication tolerance and promotion of immune evasion [ 28 - 30 ], making biofilm formation an important virulence factor of Candida species and a cause of treatment failure [ 31 , 32 ].

In its quest to focus on the increasing burden of antimicrobial resistance and to guide and promote research to improve diagnostic and treatment strategies, the World Health Organization categorized *C. albicans* and *C. glabrata* as critical and high-priority pathogens, respectively, in its first published fungal pathogen priority list [ 33 ]. Development of antifungal medication resistance in *C. albicans* and NAC species poses a serious challenge [ 34 ] and creates an urgent need for the identification and development of novel antifungal agents.

Antifungal compounds of plant origin are therapeutic alternatives that can potentially be used to curb the increasing number of fungal infections [ 35 - 37 ]. South Africa is home to the richest temperate flora in the world; 11,700 out of 19,581 indigenous plant species are endemic and 5,000 species are used as medicines, with 350 species commonly traded as medicinal plants [ 38 , 39 ]. *Galenia africana*, also known as “kraalbos”, “geelbos” or “perdebos”, belongs to the Aizoaceae family and is a local medicinal plant predominantly found in the Namaqualand and Karoo areas, extending to the Eastern Cape of South Africa [ 40 ]. Hydroethanolic extracts of *G. africana* have shown antifungal activity against 14 different fungal species *in vitro* [ 41 ], with greater antifungal activity and synergy with fluconazole demonstrated with ethanolic extracts against planktonic *C. albicans* and *C. glabrata* isolates [ 42 ].

Impedance sensors have several applications, including the evaluation of microbial biofilm formation [ 43 , 44 ]. The xCELLigence RTCA instrument (Cat. no. 05469759001, ACEA Biosciences, USA) can detect relative changes in conductivity caused by the interaction between cells adhered to the bottom of the test plate and gold microelectrodes in the presence of an electrically conductive solution [ 44 ], allowing for continuous monitoring of the adhesion properties of cells in a non-invasive label-free manner [ 45 ]. The electrical impedance is measured and recorded by the xCELLigence software as a cell index (CI), the magnitude of which is dependent on the cell number, cell morphology and size, and the strength of cell attachment to the surface of the plate [ 45 - 47 ]. The xCELLigence system has been used to measure the biofilm formation of *C. albicans* [ 48 , 49 ] and *Candida* interspecies biofilm interactions [ 50 ]. However, its application in testing the antifungal action of medicinal plants against *Candida* has not been reported.

Therefore, the present study aimed to use real-time impedance-based technology to investigate the ability of *G. africana* to disrupt existing *C. albicans* and *C. glabrata* biofilms and prevent biofilm formation.

## Materials and Methods

### Galenia africana extract preparation

*Galenia africana* was supplied as a 20% (w/v) air-dried extract of leaves and shoots, commercially prepared (Brenn-O-Ken Pty Ltd, Wolseley, South Africa) with 60% ethanol and oven-dried under negative pressure to produce crystals. Permission to conduct research on this indigenous plant was obtained from the South African Department of Forestry, Fisheries, and the Environment (Registration Number BABS 000314). A stock solution was prepared by weighing the dried crystal extract of *G. africana* and constituting it in a 50 mL centrifuge tube containing RPMI 1640 medium (Cat. no. R6504, Sigma-Aldrich, St. Louis, MI, USA). Sterile forceps were used to crush and dissolve the extract, with intermittent vortexing. The extract was incubated at approximately 20-25 °C for 24 h. This was followed by final crushing and vortexing to allow the phytochemicals of the extract to dissolve into the media. The tubes were centrifuged for 10 min at 20,000 rpm, and the supernatant was aseptically aspirated and transferred to a 15 mL centrifuge tube. Tubes were further centrifuged at 4,000 rpm for 5 min. This process was repeated several times until a debris-free, brown-green plant tincture was observed. The tincture was filter sterilized using a 5 mL sterile syringe and a 0.22 μm filter into a 2 mL sterile microcentrifuge tube. Stock concentrations (yielding a 400 mg/mL stock solution, equivalent to a 40% (w/v) extract concentration) were stored at 4 °C.

### Candida species and culture conditions

*Candida albicans* (ATCC 90028) and *Candida glabrata* (ATCC 26512), obtained from the American Type Culture Collections (ATCC, Manassas, VA, USA) were used for the real-time analysis of adherence and subsequent biofilm formation. The isolates were revived by growth in 10 mL Sabouraud dextrose broth (Cat. no. CMO147, Oxoid, UK), followed by incubation at 37 °C for 3-5 days. Purity of growth was confirmed by microscopy and subculture of single colonies previously grown on Sabouraud dextrose agar (SDA) (Cat. no. 84088, Sigma-Aldrich, St. Louis, MI, USA) and incubated aerobically at 37 °C for 24-48 h. Species differentiation was confirmed by growth on Fluka chromogenic *Candida* identification agar (Cat. no. 94382; Sigma-Aldrich, St. Louis, MI, USA) and Oxoid chromogenic *Candida* agar (Cat. no. CM1002A; Oxoid, Hampshire, UK) at 30 °C for 24-72 h. The type strains were subcultured onto SDA plates for 24 h at 37 °C and individual colonies were picked to inoculate 7 mL of fresh, sterile yeast peptone dextrose (YPD) broth (Cat. no. Y1375, Sigma-Aldrich, St. Louis, MI, USA). A nephelometer (Cat. no. V3011, ThermoFisher Scientific, Waltham, MA, USA) was used to adjust the growth to a 0.5 McFarland standard suspension, yielding a cell suspension of approximately 1×10^6^-5×10^6^ CFU/mL. This inoculum was further diluted and optimized to a working suspension of 1:20 in YPD broth.

### Real-time monitoring of *Candida* species adhesion and disruption of 10-h preformed biofilm formation

Real-time cell adhesion monitoring and subsequent biofilm assays were performed using the xCELLigence real-time cell analysis-dual purpose (RTCA-DP) instrument (Cat. no. 05469759001, ACEA Biosciences, USA). Due to the high cost of the xCELLigence gold electrode-embedded plates, experiments were performed in duplicate, with the test repeated in cases of discrepancy. The YPD broth optimized working suspension was used as the culture medium for the growth of the isolates in the E-plate 16 (Cat. no. 0546830001, ACEA Biosciences, USA), as previously described [ 49 ], since this medium is known to promote *Candida* adherence and biofilm formation [ 50 , 51 ]. The E-plates were prepared by adding fresh, sterile YPD broth (100 µL) to each well. The plates were left at 20-25 °C for 30 min to achieve equilibrium between the culture media and the E-plate surface. After equilibration of the instrument at 37 ºC and the E-plates at 20-25 °C, plates were inserted into the chamber of the RTCA-DP instrument, and the RTCA software package (Cat. No. 05454433001, ACEA Biosciences, USA) was used to measure the background impedance for each well. *Candida* suspensions (equivalent to 0.5 McFarland standard) were added to the wells of the E-plates containing 100 µL of YPD broth. Each well received 50 µL of the inoculum (as this volume provides less variation in seeding density). Sterility and growth control wells were included in each plate. All the wells contained a final volume of 150 µL/well, except the sterility control well, which contained a final volume of 200 µL of YPD. The inoculated E-plates were left at 20-25 °C for 30 min to allow the cells to settle to the bottom of the wells. Sterile distilled water was added to the surrounding evaporation-control troughs, as recommended by the manufacturer. The plates were placed into the cradle of the RTCA-DP instrument, allowing the *Candida*-type strains to enter the exponential growth phase, at approximately 10 h. An experimental procedure was logged on the RTCA software package, with impedance readings set to take place at 15-minute intervals for 10 h. Following the incubation step, the E-plates were removed from the instrument, followed by the addition of various concentrations of *G. africana*, with the final concentrations within the inoculated wells ranging from 0.78 mg/mL to 50 mg/mL. Subsequently, 50 µL of the plant extract was added to the designated wells, bringing the final volume to 200 µL. The plates were returned to the instrument and further incubated at 37 °C with impedance recorded every 15 min for an additional 38 h, bringing the final incubation time to 48 h.

### Real-time monitoring of *Candida* spp. adhesion and prevention of initial biofilm formation

Ability of *G. africana* to prevent *Candida* species adhesion and subsequent biofilm formation was evaluated using impedance-based technology, employing the same preliminary and optimization steps. However, after the equilibration steps, the wells of the E-plates were inoculated with 50 µL of various concentrations of the *G. africana* extract, ranging from 0.78 mg/mL to 50 mg/mL, followed by the addition of 50 µL of the optimized fungal suspension. The control wells were treated as described above. The E-plates were placed into the cradle of the RTCA-DP instrument and incubated for a total of 48 h at 37 °C, with impedance measured every 15 min.

## Results

### Disruption of 10-hour preformed Candida albicans adhesion and subsequent biofilm formation

Prior to the determination of the critical time point at which the adhered fungal cells would be challenged with the extract (e.g., the window of treatment of preformed *Candida* species biofilms) [ 45 ], cellular behavior and optimized medication treatment times were observed. During the initial stage of the CI curves, the *Candida* species entered a biofilm formation exponential phase at approximately 7 h, followed by entry into the mid-exponential growth phase at approximately 10 h, where CI values increased, representing adhesion and subsequent biofilm formation. *Candida* biofilm formation increased exponentially up to 15 h. Therefore, *G. africana* crude extract was administered at approximately 10 h post-seeding of the *Candida*-type strains, at the start of the mid-exponential growth phase.

**Figure 1:**
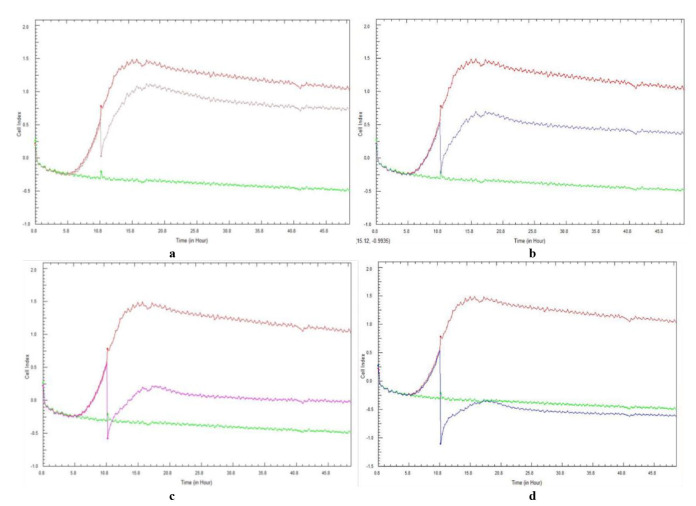
Real-time cell analysis cell index variations of 10-hour preformed *Candida albicans* (ATCC 90028) cell adhesion and subsequent biofilm formation treated with ascending concentrations of *Galenia africana* aqueous extract: a) 6.25 mg/mL, b) 12.5 mg/mL, c) 25 mg/mL, and d) 50 mg/mL. A positive control (burgundy curve) and sterility control (green curve) are included.

**Figure 2:**
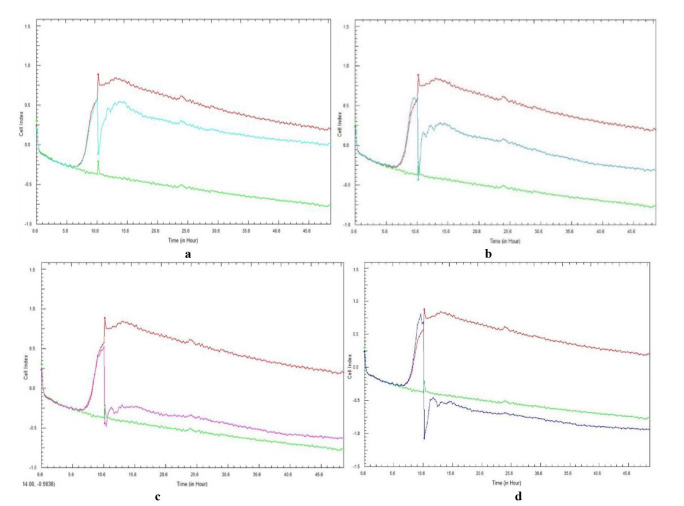
Real-time cell analysis cell index variations of 10-hour preformed *Candida glabrata* (ATCC 26512) cell adhesion and subsequent biofilm formation treated with ascending concentrations of *Galenia africana* aqueous extract: a) 6.25 mg/mL, b) 12.5 mg/mL, c) 25 mg/mL, and d) 50 mg/mL. A positive control (burgundy curve) and sterility control (green curve) are included.

Figures 1 and 2 represent the mean CI curves obtained for the duplicate experiments of the same biological sample, following a 48-h total incubation period. The curves followed the same pattern for each repeated experiment, with no discrepancies observed. In the present study, a plateau phase was observed at approximately 20 h post-incubation. The CI profile revealed that among the two *Candida* spp. tested, *C. albicans* demonstrated a stronger adherence and subsequent biofilm formation, compared to *C. glabrata*. A comparison of CI values of the positive (untreated) controls of the two Candida species revealed that *C. albicans* had a maximum CI of 1.50 at approximately 15 h and *C. glabrata* had a maximum CI of 0.89 at approximately 10 h.

When testing *C. albicans* with *G. africana*, the 6.25 mg/mL concentration (Figure 1a) resulted in a noticeable decrease in CI (0.06) after the addition of the extract at 10 h, compared to the positive control (0.7). This was observed by an immediate and transient decrease in the CI value. However, this concentration was incapable of completely eradicating or disrupting the adhesion and biofilm formation as the CI values remained positive at the end of the incubation period. Higher concentrations of *G. africana*, namely, 25 and 50 mg/mL (Figure 1c and d), proved to be the most effective in completely disrupting and removing the adhesion and biofilm formation. The CI values, -0.58 and -1.10, were the lowest values after the addition of *G. africana* extract at 10 h, in that order, compared to the CI of the growth control, with the 50 mg/mL concentration showing complete removal of the adhesion and biofilm formation. These results indicated that no adhesion and subsequent biofilm formation were present, with CI values of 0.0 and -0.5, at the end of the experiment.

As with *C. albicans*, the lower concentrations of *G. africana* did not appear to significantly disrupt the biofilm formation of *C. glabrata*, as adhesion was still evident with positive CI values, similar to the positive control at the end of the experiment. However, the 6.25 mg/mL *G. africana* concentration (Figure 2a) resulted in a noticeable decrease in CI value (-0.13) after the addition of the extract at 10 h, compared to the positive control (0.9). This was also observed in the other concentrations, with an immediate and transient decrease in the CI value. Similar to the results obtained for *C. albicans*, the lowest concentration was incapable of completely eradicating or disrupting the adhesion and biofilm formation, as the CI values remained positive at the end of incubation, while the higher concentrations of *G. africana* tested, namely 25 and 50 mg/mL (Figure 2c and d), proved to be the most effective in completely disrupting and removing the adhesion and biofilm formation.

### Prevention of *Candida* species adhesion and biofilm formation

**Figure 3:**
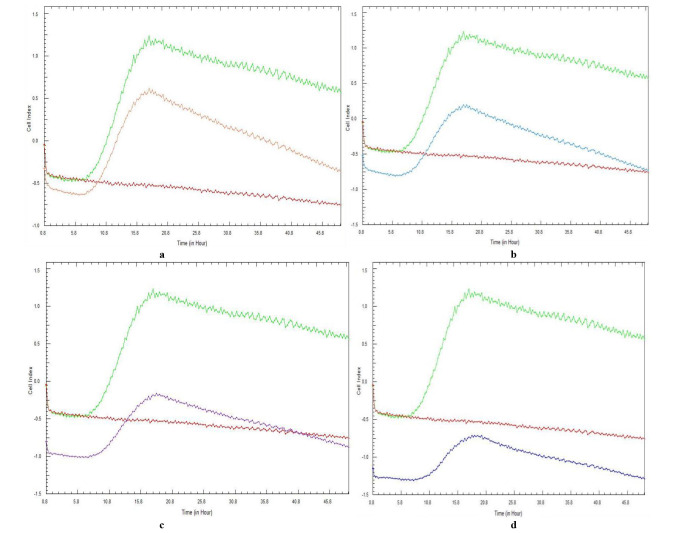
Real-time cell analysis cell index variations of *Candida albicans* (ATCC 90028) cell adhesion and subsequent biofilm formation in the presence of various concentrations of *Galenia africana* aqueous extract: a) 6.25 mg/mL, b) 12.5 mg/mL, c) 25 mg/mL, and d) 50 mg/mL. A positive control (green curve) and sterility control (burgundy curve) are included.

**Figure 4:**
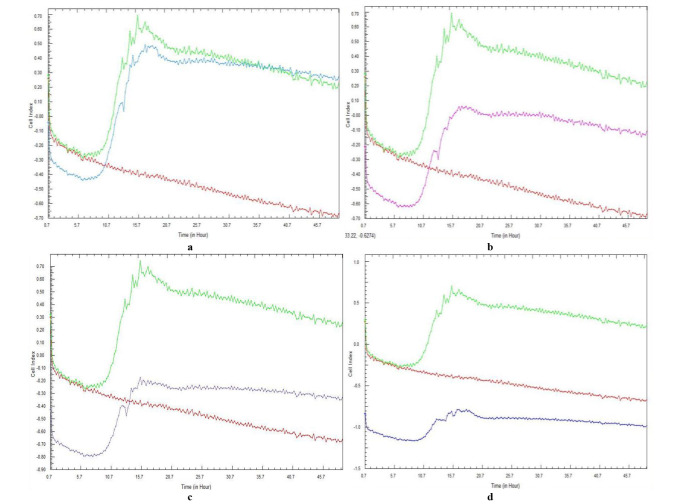
Real-time cell analysis cell index variations of *Candida glabrata* (ATCC 26512) cell adhesion and subsequent biofilm formation in the presence of various concentrations of *Galenia africana* aqueous extract: a) 6.25 mg/mL, b) 12.5 mg/mL, c) 25 mg/mL, and d) 50 mg/mL. A positive control (green curve) and sterility control (burgundy curve) are included.

Figures 3 and 4 represent the CI curves following a 48-h total incubation period. The curves followed the same pattern for each repeated experiment, with no discrepancies observed. The real-time curves relate to the ability of each concentration of *G. africana* to prevent the initial adhesion of *C. albicans* and *C. glabrata* and subsequent biofilm formation and also represent the mean CI value of duplicate experiments of the same biological sample.

The two lower concentrations of *G. africana* did not result in the immediate prevention of *C. albicans* biofilm formation. However, there was a noticeable CI reduction in these concentrations when compared to the growth control, at approximately 18 h (Figure 3a and b). When testing the 12.5 mg/mL (Figure 3b) concentration, the treated cells reached a maximum CI value of < 0.25 at approximately 18 h, after which adhesion steadily decreased. At the 25 mg/mL and 50 mg/mL *G. africana* concentrations (Figure 3c and d), *C albicans* maintained negative CI values.

When testing *C. glabrata*, the lowest concentration of *G. africana* (Figure 4a) did not appear to prevent adhesion and biofilm formation, as adhesion was still evident with CI values similar to the positive control. Unlike *C. albicans*, in which an effect on the adhesion and CI value was observed at a *G. africana* concentration of 6.25 mg/mL, an effect on *C. glabrata* adhesion was only observed at a concentration of 12.5 mg/mL, where a significant difference to the control was observed. Concentrations of *G. africana* within the range of 12.5-50 mg/mL (Figure 4b-d) proved to be the most effective in reducing the CI values, while the 25 and 50 mg/mL concentrations of *G. africana* (Figure 4c and d) maintained negative *C. glabrata* CI values. The changes in the maximum CI values obtained for both the 10-h preformed biofilm disruption and biofilm prevention demonstrated a dose-dependent response of both Candida species to the *G. africana* extract, with the *C. albicans* maximum CI values being higher than those of *C. glabrata* (Table 1).

**Table 1:** Maximum cell index values of preformed *Candida albicans* and *Candida glabrata* biofilm disruption and biofilm prevention by Galenia Africana

10-hour preformed biofilm disruption maximum cell index values (after *G. africana* extract addition)					
*Galenia africana* concentration	0 mg/mL	6.25 mg/mL	12.5 mg/mL	25 mg/mL	50 mg/mL
*Candida albicans*	1.5	1.12	0.68	0.2	-0.35
*Candida glabrata*	0.89	0.55	0.25	-0.2	-0.45
Difference	0.61	0.57	0.43	0.4	0.1
**Cell adhesion/biofilm prevention maximum CI values**					
*Galenia africana* concentration	0 mg/mL	6.25 mg/mL	12.5 mg/mL	25 mg/mL	50 mg/mL
*Candida albicans*	1.24	0.62	0.2	-0.15	-0.72
*Candida glabrata*	0.69	0.49	0.06	-0.18	-0.78
Difference	0.55	0.13	0.14	0.03	0.06

Since this was a preliminary study showing the potential antibiofilm activity of *G. africana* extract against two *Candida* species, no statistical analysis was performed.

## Discussion

This study aimed to monitor and assess the anti-biofilm properties of a *G. africana* ethanolic extract against the biofilm formation of *C. albicans* (ATCC 90028) and *C. glabrata* (ATCC 26512) in real-time, while simultaneously assessing the ability of the xCELLigence RTCA DP system to be used as a tool for screening novel medicinal plants. To the best of our knowledge, this is the first reported study to use an impedance-based system to monitor the real-time effects of a novel medicinal plant on *Candida* biofilm formation.

Although the xCELLigence system has been validated as an investigative tool for multiple complex cellular behaviors, medication responses [ 53 - 55 ], and monitoring of microbial biofilm formation [ 46 , 56 ], its use in real-time biofilm disruption using herbal extracts has not been previously documented.

There has been an increasing interest in the real-time monitoring of biofilm formation, moving away from traditional, end-point methods, such as crystal violet staining, the 2,3-bis(2-methoxy-4-nitro-5-sulfophenyl)-5-[phenylamino)carbonyl]-2H-tetrazolium hydroxide reduction assay, dry cell weight and viable cell counts, as well as molecular methods, such as DNA and protein quantification [ 57 - 60 ]. End-point methods allow for the observation or measurement of the final effect but they are labor intensive and require invasive sampling, with long interludes between sampling and obtaining a result. Conversely, the RTCA system monitors adhesion and biofilm formation in real time [ 56 ], giving a more in-depth understanding of each phase of biofilm formation [ 46 ]. In principle, the CI values increase as the biofilm adheres and proliferates on the bottom of the E-plate wells, with a peak CI being consistent with the start of biofilm maturation and a decrease occurring at the start of the biofilm detachment phase [ 56 ]. A declining slope after reaching the peak CI value has been suggested as a measure of biofilm formation [ 56 , 61 ], with further declining numbers associated with the death phase [ 62 ].

By allowing the biofilm of each *Candida* species to become established and subsequently treating them with varying concentrations of *G. africana*, biofilm disruption was achieved, although at relatively high concentrations (50 mg/mL for *C. albicans* and 25 mg/mL for *C. glabrata*). The response to *G. africana* was immediate and transient, as revealed by the precise timing and magnitude of the response by the xCELLigence system. This response could be due to the higher volume of flavanols/pinocembrin in higher doses, as they are the most active molecules within the *G. africana* extract [ 63 , 64 ].

In conventional *in vitro* assays, treatments are usually executed at predetermined time points that are convenient (e.g., 12 or 24 h post-seeding, depending on the cell type), rather than on experimental or behavioral data from the cells [ 45 ]. By contrast, the xCELLigence CI curve data revealed real-time information related to the behavior, growth, and overall health of the cells, which can be used as a guide to improve the experimental design [ 45 ].

Biofilm formation can be prevented by inhibition of its establishment [ 65 ]. In the present study, the lowest concentration of *G. africana* did not appear to prevent the adhesion and biofilm formation of *C. albicans*, as adhesion was still evident after the administration of 12.5 mg/mL extract with a decreased CI that remained negative after 24-hour incubation, while the two higher concentrations successfully inhibited *C. albicans* biofilm formation for the duration of the 48-hour incubation. In the case of *C. glabrata*, there was a short period of adhesion at a 12.5 mg/mL extract exposure, albeit at much lower CI values than *C. albicans*. This difference could be attributed to *C. albicans* being more strongly resistant in a sessile community, compared to a planktonic state [ 66 ]. Specific adhesins allow firm attachment of the microorganisms [ 67 ] and facilitate the formation of biofilms; therefore, according to the biofilm eradication and prevention curves, *C. albicans* had more adhesion potential than *C. glabrata*. The two higher concentrations completely prevented adhesion and subsequent biofilm formation for the duration of the 48-hour incubation period.

Khun et al. [ 68 ] found that *C. albicans* produced more biofilm, compared to NAC species, while Marak and Dhanashree [ 69 ] reported greater biofilm formation with *C. parapsilosis* (100%), *C. tropicalis* (61.53%), and *C. krusei* (55.55%), compared to *C. albicans* (39.02%), with *C. glabrata* showing no biofilm production. These studies support the adhesion capability and subsequent biofilm formation of *C. albicans* and *C. glabrata* in the present study, even though the methods and strains were different.

A previous study of biofilm formation of three *C. albicans* strains (one producing widespread biofilms and two mutant strains lacking adhesins) using several end-point techniques suggested that *in vitro* experiments may not accurately reflect *in vivo* realities [ 60 ]. Therefore, a comparison of strong biofilm-producing *Candida* cells and mutant strains defective in producing biofilms is desirable and overcomes a limitation of the current study.

## Conclusion

The authors are confident that the study objectives were achieved since the xCelligence RTCA could demonstrate the ability of *G. africana* to prevent the formation of *Candida* biofilms and disrupt existing *Candida* biofilms.

Further studies using the the xCelligence RTCA could explore the antimicrobial effects that individual parts of the *G. africana* plant exhibit in susceptibly assays. It would also be beneficial to test other medication-resistant *Candida* species with various adherence properties, such as *C. krusei*. Studies investigating the protein expression of medicinal plants and correlating it with their mode of action are warranted before the elucidation of gene expression data.
